# Factors associated with nursing workload in patients undergoing extracorporeal membrane oxygenation: a retrospective cohort study

**DOI:** 10.31744/einstein_journal/2025AO1743

**Published:** 2025-09-11

**Authors:** André Lucas da Silva Guideli, Luana Letícia Ribeiro de Luna, Larissa Fernandes Araújo da Silva, Marcele Liliane Pesavento, Lilia de Souza Nogueira, Filipe Utuari de Andrade Coelho

**Affiliations:** 1 Hospital Israelita Albert Einstein Critical Patients Department São Paulo SP Brazil Critical Patients Department, Hospital Israelita Albert Einstein, São Paulo, SP, Brazil.; 2 Hospital Israelita Albert Einstein Faculdade Israelita de Ciências da Saúde Albert Einstein São Paulo SP Brazil Faculdade Israelita de Ciências da Saúde Albert Einstein, Hospital Israelita Albert Einstein, São Paulo, SP, Brazil.; 3 Universidade de São Paulo Escola de Enfermagem Department of Medical-Surgical Nursing São Paulo SP Brazil Department of Medical-Surgical Nursing, Escola de Enfermagem, Universidade de São Paulo, São Paulo, SP, Brazil.

**Keywords:** Critical care nursing, Workload, Extracorporeal membrane oxygenation, Critical care, Nursing staff, Intensive care units

## Abstract

**Objective:**

To identify the factors associated with nursing workload with patients undergoing extracorporeal membrane oxygenation at 24 hours after initiation, as quantified using the Nursing Activities Score.

**Methods:**

This retrospective cohort study was conducted at an Extracorporeal Life Support Organization center in São Paulo, Brazil, and included adult patients who underwent extracorporeal membrane oxygenation between 2012 and 2023. Nursing workload was assessed using the Nursing Activities Score at intensive care unit admission and during the initial 24 and 48 hours of support. A linear regression model was used to identify factors associated with nursing workload during the initial 24 hours of support.

**Results:**

A total of 155 patients were included, with a mean age of 50.8±14.6 years, and 68.1% were male. The mean Nursing Activities Score at intensive care unit admission was 91.7%, increasing to 139.8% in the initial 24 hours of support, and slightly decreasing to 126.9% at 48 hours. The main factors associated with increased nursing workload during the initial 24 hours of extracorporeal membrane oxygenation were the presence of coronavirus disease 2019 (Estimate: 7.112; 95% confidence interval [95%CI]= 2.12-4.43; p=0.005) and the duration between intensive care unit admission and support initiation (Estimate: 2.890; 95%CI= 4.94-8.40; p=0.006).

**Conclusion:**

The nursing workload in the initial 24 hours of support was significantly elevated, influenced by clinical factors such as coronavirus disease 2019 and delayed support initiation. These findings highlight the need for structured care protocols and adequate nursing staff allocation to optimize patient outcomes and reduce excessive workload in extracorporeal membrane oxygenation management.

## Highlights

■Nursing workload is high with patients undergoing extracorporeal membrane oxygenation.

■The initial 24 hours of extracorporeal membrane oxygenation have the highest workload, quantified using the Nursing Activities Score.

■Presence of COVID-19 and delayed extracorporeal membrane oxygenation initiation increase the workload in the initial 24 hours of extracorporeal membrane oxygenation.

■Extracorporeal membrane oxygenation care demands adjustments in the nurse-to-patient ratio in the intensive care unit.

## In Brief

Nursing workload with patients receiving extracorporeal membrane oxygenation was significantly elevated, peaking in the initial 24 hours of support. Presence of COVID-19 and delayed initiation of extracorporeal membrane oxygenation were key factors in this increase, emphasizing the need for staffing strategies tailored for high-complexity intensive care scenarios.

## Graphical abstract

**Figure:**
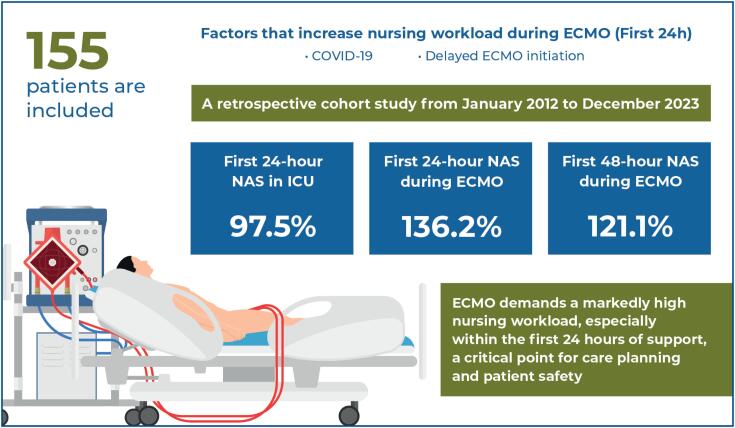

## INTRODUCTION

Extracorporeal membrane oxygenation (ECMO) is an advanced life-support system used in critically ill patients with severe cardiac or respiratory failure, which is refractory to conventional treatment and requires prolonged extracorporeal circulation and gas exchange to maintain adequate oxygenation and hemodynamic stability.^(1)^ Given its complexity, ECMO requires continuous monitoring and specialized multidisciplinary care, with nurses playing a central role in ensuring patient safety, circuit integrity, and complication prevention.^(2-4)^ The management of patients on ECMO is a substantial nursing workload, as nurses must integrate advanced clinical reasoning with highly specialized interventions to optimize patient outcomes.^(5,6)^

To enhance oxygenation and support organ function, ECMO is classified into two main configurations: venovenous (VV) and venoarterial (VA).^(1,2)^ Venovenous ECMO is primarily indicated in severe respiratory failure in which conventional strategies have proven insufficient.^(1,2)^ In contrast, VA ECMO is used in cardiac failure, particularly cardiogenic shock, septic shock, toxicological emergencies, or challenges related to weaning from cardiopulmonary bypass in the perioperative setting.^(1,2)^

Extracorporeal membrane oxygenation has been used clinically since the 1970s, and its global adoption has markedly increased over the past two decades, particularly in response to two major public health crises: the 2009 H1N1 influenza outbreak and, the more recent, the coronavirus disease 2019 (COVID-19) pandemic.^(7)^ Both diseases were characterized by severe pulmonary complications, necessitating advanced respiratory support and reinforcing the role of ECMO as a lifesaving intervention.^(7)^

According to the Extracorporeal Life Support Organization (ELSO), the current ECMO decannulation rate is approximately 67%, with an overall hospital survival rate of 54%. Neonatal patients achieve higher survival rates than adult patients.^(8)^ Despite the improved outcomes, ECMO remains a highly complex and resource-intensive therapy that demands specialized training and continuous multidisciplinary management involving perfusionists, intensivists, physiotherapists, and nurses to ensure patient safety and optimal outcomes.^(2,9)^

Among healthcare professionals involved in ECMO management, nurses play an essential role in direct patient care.^(2)^ Nursing care is essential in all stages of ECMO support, from the moment of indication and cannulation, through the management of material resources and ensuring procedural safety, to the maintenance phase, which involves system monitoring and complication reduction related to both the circuit and the patient, and finally, during decannulation.^(9,10)^ Given the specialized nature of these interventions, nursing professionals must possess a deep clinical understanding of the ECMO pathophysiology, anticipate complications, and promptly prevent life-threatening events.^(9,10)^

Recognizing this demand, the Brazilian Regional Nursing Council (COREN - *Conselho Regional de Enfermagem do Brasil*) established Resolution No. 33/2011, which mandated that only nurses who have completed a specific ECMO training are qualified to provide direct care to these patients.^(11)^ This regulation defines ECMO nursing as an advanced practice, underscoring the need for specialized education and competencies. However, it does not formally specify nurse-to-patient ratios during ECMO support, despite professional recommendations suggesting the adoption of a 1:1 ratio from the moment ECMO is initiated.^(2,4,5)^ This is because the highly complex nature of ECMO management demands continuous monitoring, rapid clinical intervention, and coordinated multidisciplinary collaboration, which inherently contribute to a consistently high nursing workload.^(12,13)^

The Nursing Activities Score (NAS) is the most widely used instrument for quantifying nursing workload in intensive care units (ICUs). It consists of 23 items of essential nursing activities, including basic activities, ventilatory support, cardiovascular support, renal support, neurological support, metabolic support, and specific interventions.^(13,14)^ Although the NAS has been extensively applied in the care of critically ill patients, studies on nursing workload, specifically with patients undergoing ECMO, remain scarce.

A retrospective Italian study found that the NAS with patients on ECMO was significantly higher than with patients not on ECMO (87.0% *versus* 67.2%).^(13)^ Similarly, a recent Brazilian study showed that nursing workload peaked at ICU admission and further increased on the day of ECMO initiation (105.0% *versus* 126.0%).^(15)^

These findings reinforce the substantial increase in care demand during the early phase of ECMO support. The period of initial 24 hours following ECMO initiation is critical and characterized by hemodynamic instability and need for intensive monitoring and frequent interventions related to circuit maintenance and parameter adjustment.^(2,4,5,15)^ However, to date, no studies have specifically quantified nursing workload during this initial 24-hour period or investigated the clinical and organizational factors that contribute to its intensification.^(2,4,5,15)^ Moreover, the nursing workload tends to remain high even beyond the initial 24 hours because of the sustained complexity of ECMO-related care.^(15)^

Addressing this knowledge gap is essential for optimizing nursing staff allocation, enhancing patient safety, and improving ICU resource management. By identifying the determinants of nursing workload after ECMO initiation, training strategies, staffing models, and evidence-based planning can be formulated to ensure high-quality care in highly complex settings.

## OBJECTIVE

To identify the factors associated with nursing workload, as quantified using the Nursing Activities Score, with patients undergoing extracorporeal membrane oxygenation, in the initial 24 hours after extracorporeal membrane oxygenation initiation.

## METHODS

### Study design

This retrospective cohort study followed the Strengthening the Reporting of Observational Studies in Epidemiology recommendations for observational research.^(16)^

### Setting

This study was conducted in an ICU of a private quaternary care hospital in São Paulo, Brazil. The hospital has 706 beds, of which 37 are allocated to the adult medical-surgical ICU. The hospital is an ELSO center with a multidisciplinary team (including physicians, nurses, and physiotherapists) trained to provide specialized ECMO care. The nurse-to-patient ratio after ECMO initiation was maintained at 1:1, ensuring that the nurses delivered comprehensive care to both the patient and the ECMO circuit, with the nurses responsible for this care being specifically trained in ECMO support.

### Study participants

Patients aged >18 years who were admitted to the ICU and underwent ECMO between 2012 (beginning of ECMO support at the hospital) and 2023 were included in the study. Patients with ECMO duration <24 hours were excluded.

### Data collection and study variables

Patient data were extracted from the Epimed Monitor System^®^ platform (Epimed Solutions, Rio de Janeiro, Brazil), which is a structured electronic system in which data related to patients admitted to the ICU are entered.^(17)^ After extraction, the data were entered into the Research Electronic Data Capture^®^ (REDCap^®^) platform for data security, anonymization, and subsequent analysis.^(18)^

Although the NAS was recorded at three time points, 24 hours after ICU admission and 24 and 48 hours after ECMO initiation, only the NAS at 24 hours after ECMO initiation was used for the association analyses. The NAS at other time points was recorded exclusively for descriptive purposes, to characterize the trajectory of nursing workload throughout patient's clinical course.

The NAS quantifies nursing workload, and the score is the sum of points expressed as a percentage.^(19)^ This instrument consists of 23 items related to nursing care, divided into seven categories, each item is scored on a scale of 1.2-32.0 points, with the maximum total score of 176.8%. Each point on the NAS corresponds to 14.4 minutes of nursing care.^(19)^ The NAS was retrieved from patients’ electronic health records, which was calculated and documented daily by bedside nurses trained in the standardized use of this instrument to ensure accurate and consistent assessment of nursing workload.

To characterize the study sample, the following demographic and clinical variables were collected: sex, age, and comorbidities. Clinical conditions on the day of ECMO cannulation were also recorded, including the presence of pressure injury (PI), incontinence-associated dermatitis (IAD), use of neuromuscular blockers, prone positioning, intra-aortic balloon pump (IABP) use, and renal replacement therapy (RRT). To assess clinical severity and organ dysfunction, the Simplified Acute Physiology Score 3 (SAPS 3), calculated in the first hour of ICU admission, and the Sequential Organ Failure Assessment (SOFA) score, obtained at ICU admission, were recorded.^(20,21)^ Extracorporeal membrane oxygenation-related variables included modality, cannulation details, ECMO duration, and duration between ICU admission and ECMO initiation. Respiratory ECMO Survival Prediction and Survival After Veno-Arterial ECMO scores were calculated at the start of ECMO.^(22,23)^ The Vasoactive-Inotropic Score (VIS), calculated at ECMO initiation, was also included to quantify cardiovascular support.^(24)^

The variables considered for association analyses were sex, COVID-19 diagnosis, IABP use, neuromuscular blocker use, prone positioning, PI, IAD, RRT, SAPS 3, SOFA score, age, VIS, mechanical ventilation (MV) duration, and duration between ICU admission and ECMO initiation. Data pertaining to resource utilization variables, such as ICU and hospital length of stay and hospital mortality, were also collected but were not included in the association analyses.

### Statistical analysis

For the analyses, the data entered in the REDCap^®^ platform were imported in an electronic spreadsheet (Microsoft^®^ Windows Excel-2007). ‘Statistical Package for the Social Sciences’ (SPSS), version 26 was used for analyses. The descriptive analysis for qualitative variables was based on absolute and relative frequencies. For quantitative variables, the type of distribution was tested using the Shapiro-Wilk test, and after confirming normality, the means and standard deviations were calculated.

For the analysis of categorical variables, the Student's *t*-test was used, and for continuous variables, Pearson's correlation test was employed. To compare the NAS in the initial 24 hours in the ICU with the NAS in the initial 24 and 48 hours on ECMO, analysis of variance (ANOVA) was used. All variables that showed a correlation with NAS with a p≤0.2 were selected to be analyzed in the model.^(25)^ A linear model was used in the analysis of NAS to identify factors associated with nursing workload in the initial 24 hours of ECMO initiation. Multicollinearity among the independent variables was assessed using the Variance Inflation Factor (VIF), the model's goodness of fit was evaluated using the coefficient of determination (R²), and the strength and direction of associations were described using beta coefficient (β) and the respective standard error (SE). Values with p≤0.05 were considered statistically significant for all the conducted analyses.

### Ethical considerations

This study was approved by the Ethics Committee of *Hospital Israelita Albert Einstein* (CAAE: 80502324.8.0000.0071; #5.238.293). The requirement for informed consent was waived by the ethics committee.

## RESULTS

A total of 164 patients were analyzed; however, only 155 were included, with nine being excluded because of ECMO duration <24 hours. Table 1 presents the demographic, clinical, and ECMO-related characteristics of patients undergoing ECMO. Most patients were male, with a mean age of 49.8±15.9 years and the most prevalent comorbidity of systemic hypertension. MV and noradrenaline were required in almost all patients. The mean VIS was 121.5±248.2, and more than half of the patients required RRT. Venovenous ECMO was the most frequently used modality, and peripheral cannulation was the predominant approach with mean ECMO duration of 13.0±18.0 days and duration between ICU admission and ECMO initiation of 6.3±15.4 days. More than half of the patients experienced hospital mortality and decannulation. The mean SAPS 3 and SOFA score were 50.2±13.9 and 9.0±3.6, respectively.

**Table 1:** Demographic, clinical, and extracorporeal membrane oxygenation-related characteristics of patients

Variable		
Sex, n (%)		
	Male	104 (67.1)
Age (years), mean±SD		49.8±15.9
Comorbidities, n (%)		
	Systemic hypertension	50 (32.3)
	Heart failure	42 (27.1)
	Diabetes mellitus	34 (21.9)
	Chronic obstructive pulmonary disease	22 (14.2)
	Chronic kidney disease	10 (7.7)
Vasopressors, n (%)		
	Noradrenaline	154 (99.4)
	Adrenaline	80 (51.6)
	Dobutamine	72 (46.0)
	Vasopressin	39 (25.2)
	Milrinone	27 (17.4)
Vasoactive inotropic score, mean±SD		121.5±248.2
Mechanical ventilation, n (%)		154 (99.4)
ICU LOS (days), mean±SD		19.3±33.8
Hospital LOS (days), mean±SD		29.3±29.7
Renal replacement therapy, n (%)		100 (64.5)
Simplified Acute Physiology Score 3, mean±SD		50.2±13.9
Sequential Organ Failure Assessment score, mean±SD		9.0±3.6
Death, n (%)		89 (57.4)
ECMO modality, n (%)		
	Venovenous	81 (52.3)
	Venoarterial	76 (49.0)
Cannulation, n (%)		
	Periphery	140 (90.3)
	Central	16 (10.3)
ECMO duration (days), mean±SD		13.0±18.0
Duration between ICU admission and ECMO initiation (days), mean±SD		6.3±15.4
Respiratory ECMO Survival Prediction score, mean±SD		-1.7±2.5
Survival After Veno-Arterial ECMO score, mean±SD		-0.4±3.5
Decanulation, n (%)		101 (65.2)

SD: standard deviation; ICU: intensive care unit; LOS: length of stay; ECMO: extracorporeal membrane oxygenation.

**Figure 1:**
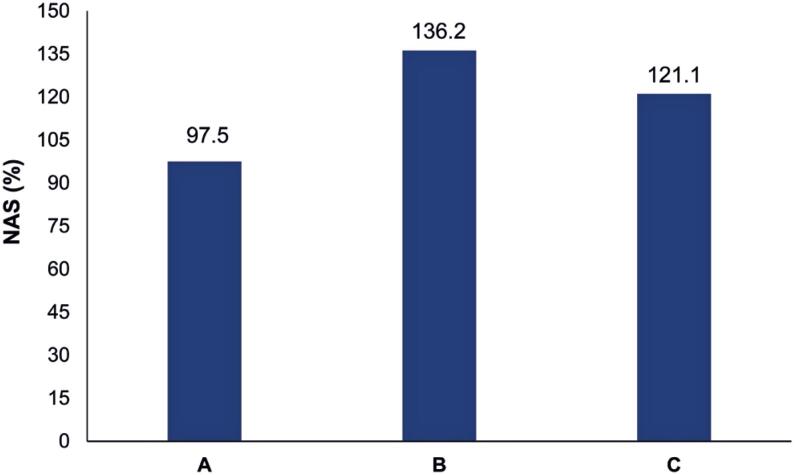
Changes in the Nursing Activities Score during the initial 24 hours in the intensive care unit and the initial 24 and 48 hours on extracorporeal membrane oxygenation

Figure 1 illustrates the changes in the NAS during the initial 24 hours in the ICU and the initial 24 and 48 hours of ECMO support. The data show a significant increase in the NAS between ICU admission and ECMO initiation, followed by a slight reduction after 48 hours of ECMO. The results of ANOVA revealed that the NAS was significantly different between the initial 24 hours in the ICU and the initial 24 and 48 hours on ECMO (p=0.003 and p=0.042, respectively).

A: NAS during the initial 24 hours in the ICU; B: NAS during the initial 24 hours on ECMO; C: NAS during the initial 48 hours on ECMO.

The results of bivariate analysis for demographic and clinical variables that affect the NAS in the initial 24 hours of ECMO are presented in table 2.

**Table 2:** Bivariate analysis for demographic and clinical characteristics affecting the Nursing Activities Score in the initial 24 hours on extracorporeal membrane oxygenation in patients undergoing extracorporeal membrane oxygenation

Variable		24-hour ECMO NAS			p value
		n	Mean	Standard deviation	
Sex					0.883#
	Male	104	136.4	21.3	
	Female	51	135.9	20.7	
Coronavirus disease 2019 diagnosis					0.008#
	Yes	52	142.3	18.5	
	No	103	132.9	21.3	
Neuromuscular blocker use					
	Yes	103	134.8	21.6	0.295#
	No	52	138.5	19.2	
Prone positioning					0.079#
	Yes	14	145.4	15.0	
	No	141	135.4	21.1	
Incontinence-associated dermatitis					0.338#
	Yes	12	141.8	20.9	
	No	141	135.8	16.0	
Pressure injury					0.317#
	Yes	27	132.6	19.9	
	No	126	132.9	23.9	
Intra-aortic balloon pump use					0.575#
	Yes	39	136.5	26.2	
	No	116	134.7	18.8	
Renal replacement therapy					0.955#
	Yes	100	136.1	20.8	
	No	55	135.9	21.2	

# Student's *t-*test.

NAS: Nursing Activities Score; ECMO: extracorporeal membrane oxygenation.

Table 3 presents the correlation between the NAS in the initial 24 hours on ECMO and quantitative demographic and clinical variables in patients undergoing ECMO. Notably, only duration between ICU admission and ECMO initiation was positively correlated with the NAS in the initial 24 hours on ECMO.

**Table 3:** Correlation between Nursing Activities Score in the initial 24 hours on extracorporeal membrane oxygenation and quantitative demographic-clinical variables in patients undergoing extracorporeal membrane oxygenation

Variable	24-hour ECMO NAS	
	r*	p value
Age	0.107	0.183#
Sequential Organ Failure Assessment score	0.637	0.144#
Simplified Acute Physiology Score 3	0.070	0.389#
Vasoactive-Inotropic Score	0.048	0.555#
Duration between intensive care unit admission and ECMO initiation	0.794	0.029#
Mechanical ventilation duration	0.044	0.589#

* Pearson's correlation coefficient;

# Pearson's correlation test.

NAS: nursing activity score; ECMO: extracorporeal membrane oxygenation.

Table 4 presents a linear regression model assessing the factors associated with workload, as quantified using the NAS in the initial 24 hours on ECMO. Notably, the following factors were found to be significantly associated with workload: COVID-19 diagnosis (estimate: 9.932; standard error [SE]: 1.78; 95% confidence interval [95%CI]= 3.12-16.69; p=0.004) and the duration between ICU admission and ECMO initiation (estimate: 2.790; SE: 2.10; 95%CI= 4.85-9.81; p=0.008). The VIF and R² values were 1.2 and 0.478, respectively.

**Table 4:** Linear regression model assessing factors associated with workload as quantified using the Nursing Activities Score in the initial 24 hours on extracorporeal membrane oxygenation

Variable	Estimate (beta coefficient)	Standard error	95% confidence interval	p value
Coronavirus disease 2019 diagnosis	9.932	1.78	3.12-16.69	0.004
Prone positioning	5.485	4.40	-6.01-19.68	0.374
Duration between intensive care unit admission and ECMO initiation	2.790	2.10	4.85-9.81	0.008
Age	0.080	0.99	1.02-1.46	0.302
Sequential Organ Failure Assessment score	-0.669	0.36	-1.61-0.27	0.163

ECMO: extracorporeal membrane oxygenation.

## DISCUSSION

The results of this study showed that the nursing workload with patients undergoing ECMO is significantly high in the initial 24 hours after ECMO initiation. The increased workload is significantly associated with the presence of COVID-19 and the duration between ICU admission and ECMO initiation, reflecting the clinical complexity of patient care, which necessitates advanced care interventions and increases the volume of activities required for clinical stabilization.

The NAS assessment during the study period confirmed that the most critical phase of nursing workload was the initial 24 hours after ECMO initiation. Lucchini et al. demonstrated that the nursing workload is particularly high during the initiation of ECMO support because of the need for continuous monitoring, frequent parameter adjustments, and management of acute complications.^(13)^

Presence of COVID-19 and the duration between ICU admission and ECMO initiation were the most significant factors associated with increased nursing workload in the initial 24 hours on ECMO. Regarding COVID-19, a Brazilian study conducted in 2023 revealed that nursing workload, as quantified using the NAS, was significantly higher for patients with COVID-19.^(26)^ The increased demand for MV, vasopressors, sedatives, and neuromuscular blockers plays a major role in this workload increase.^(26)^ Furthermore, an analysis of specific NAS items showed that patients with COVID-19 required greater monitoring, hygiene procedures, mobilization and positioning care, managerial responsibilities for nurses, airway and MV management, and need for invasive procedures in the ICU.^(26)^

Another Italian study on the determinants of nursing workload in the context of COVID-19 also revealed higher NAS in patients with COVID-19.^(6)^ The multivariate analysis confirmed that COVID-19 was a significant factor influencing the NAS, driven by its clinical impact, which increased both the frequency and complexity of nursing care.^(6)^ Not only the clinical alterations caused by COVID-19, but also protocol-driven activities such as donning personal protective equipment, environmental decontamination, family support, and management of care-related technologies contribute to nursing workload.^(27)^

Another significant factor identified in this study that affected the nursing workload was the duration between ICU admission and ECMO initiation. A direct relationship between these variables has not been established in the literature, focusing instead on the clinical outcomes associated with delayed ECMO initiation.^(28-32)^

In a study on cardiogenic shock requiring VA ECMO, delayed ECMO initiation after its indicated timing resulted in worsening acidosis, increased need for percutaneous coronary angioplasty, and greater use of additional mechanical circulatory support devices, such as IABP and ventricular assist devices.^(28)^Similarly, a study analyzing ECMO initiation in patients undergoing tricuspid valve repair surgery found that delayed ECMO initiation led to a reduction in cardiac index, increased dependence on vasopressors and inotropes, prolonged MV duration, and higher in-hospital mortality.^(29)^

The literature on delayed VV ECMO initiation remains inconclusive regarding its precise clinical impact. However, a study that investigated a prolonged interval between ICU admission and ECMO cannulation in patients with COVID-19 reported a significant reduction in ECMO weaning success rates and a substantial increase in in-hospital mortality.^(30,31)^

Delayed ECMO cannulation leads to hemodynamic instability, increasing the need for intensive nursing interventions and continuous monitoring.^(28-31)^ This, in turn, significantly impacts the NAS, reflecting the increased demand for clinical stabilization efforts and continuous patient assessment.^(32)^ Similarly, the insertion of additional mechanical circulatory support devices and the subsequent clinical deterioration reflected in the prognostic scores contribute to a substantial increase in the nursing workload.^(33,34)^

In summary, the findings of this study provide essential insights into the nursing workload associated with ECMO support and its determinants. The importance of specialized nursing training in ECMO patient care is emphasized as the complexity of this setting requires institutions to ensure that nurses are adequately prepared to address the identified challenges, including continuous monitoring, vasoactive drug titration, and early complication detection.^(35)^ Moreover, these findings contribute to the development of ICU staffing policies, underscoring the need for a nurse-to-patient ratio that appropriately reflects the high demand of ECMO care.^(36)^

Some limitations of this study should be identified, the primary being its single-center design, which may restrict the external generalizability of the findings. Therefore, future multicenter studies are required to determine whether these results are consistent across different institutions and settings. Additionally, the retrospective nature of the study, based on data records, may have introduced biases related to missing or incomplete information. Prospective observational studies could provide more robust evidence of real-time variations in nursing workload among patients undergoing ECMO.

## CONCLUSION

This study demonstrated that the nursing workload with patients undergoing extracorporeal membrane oxygenation was significantly high, particularly during the initial 24 hours after support initiation. The main factors associated with this increase were the presence of COVID-19 and the duration between intensive care units admission and extracorporeal membrane oxygenation initiation, reflecting the clinical complexity and need for intensive care to stabilize these patients. Understanding these factors is essential to guide resource allocation, develop evidence-based staffing models, and implement targeted interventions to promote patient safety and support high-performance nursing care.
